# Maternal Respiratory Syncytial Virus (RSV) Vaccination: Current Status and Comparison to Monoclonal Antibodies (mAbs) for RSV Prevention in Infants and Children

**DOI:** 10.34763/jmotherandchild.20252901.d-25-00012

**Published:** 2025-08-16

**Authors:** Ahila Ali, Laiba Shamim, Ahmed Ibrahim, Muhammad Abdullah Humayun, Muhammad Hamza Khan, Anum Akbar, Sanmit Jindal, Shahzaib Ahmed, Jamuna Shrestha, Muhammad Abdullah Nveed

**Affiliations:** Department of Internal Medicine, Dow Medical College, Karachi, Pakistan; Department of Internal Medicine, Sindh Medical College, Karachi, Pakistan; Department of Internal Medicine, Faculty of Medicine, Alexandria University, Alexandria, Egypt; Department of Pediatrics, Shalamar Institute of Health Sciences, Lahore, Pakistan; Department of Internal Medicine, Karachi Medical and Dental College, Karachi, Pakistan; Department of Pediatrics, University of Nebraska Medical Center, Omaha, Nebraska, USA; Department of Department of Population Health and Leadership, University of New Haven, Connecticut, USA; Department of Internal Medicine, Fatima Memorial Hospital College of Medicine and Dentistry, Lahore, Pakistan; Department of Pediatrics, Manipal College of Medical Sciences, Pokhara, Nepal

**Keywords:** Respiratory syncytial virus, vaccine, monoclonal antibodies, immunization, pregnancy

## Abstract

Respiratory Syncytial Virus (RSV) causes over 50,000 hospitalizations annually among children under five years of age, leading to long-term consequences, such as asthma. Monoclonal antibodies (mAb) have been recommended for prevention, but their limitations have prompted the search for alternative preventive measures. The recent Food and Drug Administration (FDA) approval of a maternal RSV vaccine with 80% efficacy in protecting infants up to 90 days post-birth marks a significant advancement. Our narrative review investigates the differences in RSV immunization in pregnant mothers versus infants and children, with the goal of identifying factors that influence parental decisions. This study provides insights for optimising preventive strategies, and the results highlight the importance of maternal vaccination in combating RSV in children.

## 1. Introduction

Respiratory Syncytial Virus (RSV) is a prevalent cause of upper respiratory tract infections (URTI), and can progress to lower respiratory tract infections (LRTI), particularly in vulnerable groups, such as infants, immunocompromised individuals, and the elderly [1]. RSV commonly circulates seasonally in temperate regions, typically from late autumn to early spring. RSV is transmitted through respiratory droplets, as well as proximity to infected individuals or objects that have been contaminated with the virus [2]. Beyond causing immediate illness, RSV infections in infancy have been linked to persistent wheezing and the long-term development of asthma [3].

Infants often experience hospitalization from RSV infection within the first six months, and this is a leading cause of neonatal deaths worldwide [4]. Risk factors for severe RSV infection in infants include preterm delivery and cardiopulmonary disorders [5]. RSV infects almost the entire global population before the age of two, with peak hospitalization rates occurring between the second and third months of life. However, the potential for severe disease persists until approximately five years of age. Approximately 64 million people experience RSV globally, resulting in millions of hospitalizations and thousands of deaths [6]. Most children contract RSV by the age of two years, with reinfections occurring throughout life, usually resulting in mild or asymptomatic cases [7]. RSV-related LRTI, however, can lead to complications, such as respiratory distress, pneumonia, and bronchiolitis, sometimes with fatal outcomes [8].

RSV is a non-segmented RNA virus with two subtypes (RSV A and RSV B) and a genome comprising 10 genes encoding 11 proteins [9]. The G and F proteins on the virus's surface play crucial roles in its entry, replication, and immune modulation [10]. Aantiviral drugs and vaccines primarily targets the F protein, which is a major focus of neutralizing antibodies generated during infection. The G protein, expressed as either membrane-bound or soluble, significantly evades the immune response [11]. Immunization is a key approach for protecting infants and children against RSV, either whether it is passive immunization via monoclonal antibodies (mAbs) and maternal antibodies, or active immunization via RSV vaccination. This narrative review aims to critically compare various immunization methods for children against RSV, discussing the strengths, limitations, and future directions of each. It further discusses the factors influencing a parent's choice of one method over another.

## 2. Overview of studies on developing RSV vaccines for children

Vaccinating infants and children against RSV has emerged as a critical public health concern, prompting multiple vaccination trials with various iterations. The field of RSV vaccine development is primarily influenced by five key technologies, focusing on four types of active immunizing agents: live attenuated vaccines, particle-based vaccines, subunit-based vaccines, and vector-based vaccines [12]. There have been setbacks in the development of RSV vaccines, most notably the experimental formalin-inactivated vaccination that failed over 50 years ago in young children [13]. Several vaccines were considered too attenuated and inappropriate for future development, including LIDcpΔM2-2 and the chimpanzee-derived replication-deficient adenoviral vector (ChAd155-RSV) [13,14]. Various clinical trials conducted in the 1970s, 1980s, and 1990s investigated various approaches to developing an RSV vaccine in children and showed mixed results. A trial conducted in 1979–1980 evaluated a live RSV vaccine administered parenterally to children under 24 months of age and found it ineffective in preventing RSV infection during epidemics [15].

In 1997, another trial focused on the purified F protein RSV vaccine (PFP-2) among children under 12 months of age with bronchopulmonary dysplasia. It demonstrated safety, immunogenicity, and potential protection against serious RSV disease upon reinfection [16]. Another study in 1993 administered an RSV subunit vaccine primarily composed of F glycoprotein to children aged 18–36 months who had previously been hospitalized for RSV infection. It showed promising results in preventing subsequent RSV infections without significant side effects [17]. Notably, in a vaccine trial in the 1960s, infants and young children were immunized using either a formalin-inactivated whole virion RSV preparation (FI-RSV) or a comparable paramyxovirus preparation (FIPIV). Among those who received FI-PIV and later encountered natural RSV infection in the subsequent season, 5% required hospitalization. In contrast, among those immunized with FI-RSV and subsequently infected with RSV, 80% required hospitalization, resulting in the unfortunate loss of two children's lives [2,18]. Concerns have been raised about non-live vaccine formulations potentially leading to vaccine-enhanced RSV disease, similar to issues that occurred with a formalin-inactivated vaccine administered to infants without prior exposure to the virus in the 1960s [18].

The only vaccine strategies that have been shown not to trigger enhanced disease are live attenuated and live chimeric virus formulations [19]. However, the challenge lies in finding the right equilibrium between attenuation for safety, ensuring immunogenicity, and maintaining genetic stability [20, 21]. After five decades of research and development, no approved RSV vaccine for infants and children is currently available [19]. Table 1 presents a concise overview of studies focused on the development of RSV vaccines for infants and children.

**Table 1.** Studies on RSV Vaccine Development and Efficacy in Infants and Children

**Study ID**	**Type of Active Immunizing Agent**	**Population**	**Study Design**	**Conclusion**
Cunningham et al, 2020^22^	Live Attenuated Vaccine	6–24 months	Randomized control trial	Two candidate vaccines were compared: RSV/ΔNS2/Δ1313/I1314L and RSV/276. Both vaccines were well tolerated, highly infectious, and immunogenic in children, showing potential for RSV vaccination in children aged 6–24 months.
McFarland et al, 2018^23^	Live Attenuated Vaccine	6–24 months	Randomized control trial	The LID ΔM2-2 vaccine demonstrated good infectivity and immunogenicity in RSV-seronegative children, with a ≥4-fold increase in antibody levels and good protection in the subsequent RSV season.
McFarland et al, 2020^24^	Live Attenuated Vaccine	6–24 months	Randomized control trial	The LID/ΔM2-2/1030s vaccine demonstrated high immunogenicity, with 95% of participants showing a ≥4-fold increase in serum RSV-neutralizing antibody levels.
Malkin et al, 2013^25^	Live Attenuated Vaccine	6–24 months	Randomized control trial	MEDI-559 (live attenuated intranasal vaccine) showed good safety and sero-response (59% of recipients). It was well tolerated with mild and moderate side effects.
Belshe RB et al, 1982^15^	Live Attenuated Vaccine	Children under 24 months	Field trial	The live RSV vaccine administered parenterally was ineffective in preventing RSV infection during epidemics, highlighting challenges with live vaccine formulations.
Groothuis JR et al, 1998^16^	Subunit-Based Vaccine (Purified F protein)	Children under 12 months with bronchopulmonary dysplasia	Clinical trial	The purified F protein RSV vaccine (PFP-2) demonstrated safety, immunogenicity, and potential protection against severe RSV disease in children with bronchopulmonary dysplasia.
Tristram DA et al, 1993^17^	Subunit-Based Vaccine (F glycoprotein)	Children 18–36 months (seropositive for RSV)	Clinical trial	The F glycoprotein-based subunit vaccine showed promising results in preventing subsequent RSV infections in children who had previously been hospitalized for RSV without significant side effects.
Kim HW et al, 1969^18^	Inactivated Vaccine (FI-RSV)	Infants and young children	Clinical trial	Infants vaccinated with FI-RSV had a significantly higher risk of severe disease and hospitalization when later infected with RSV, leading to fatalities in two cases.
Graham BS, 2017^19^	Review (Live Attenuated, Chimeric Virus Vaccines)	N/A	Review	Live attenuated and live chimeric RSV vaccines are among the most promising candidates. Studies show that, unlike inactivated vaccine formulations, they do not trigger enhanced disease.
Tang RS et al, 2008^20^	Vector-Based Vaccine (PIV-Vectored RSV Vaccine)	Healthy adults	Preclinical and initial clinical trial	The PIV-vectored RSV vaccine was tested for safety and enhanced disease in healthy adults. It showed promise in the preclinical phase but required further investigation for broader use.
Wright PF et al, 2007^21^	Live Attenuated Vaccine	Children	Clinical trial	Live attenuated RSV vaccines did not cause enhanced disease after exposure to wild-type RSV, suggesting their potential safety in preventing RSV infections.

## 3. Current RSV immunization to infants and children

### 3.1. Passive immunization to infants and children

#### 3.1.1. Monoclonal antibodies (mAbs)

mAbs are immunizing agents that protect infants and children from RSV. Palivizumab, a monoclonal antibody (mAb) that has been authorized for over 20 years, remains the sole preventive option against RSV. However, its use is confined to a narrow segment of the pediatric population — those under 35 weeks of gestational age, and those up to six months of age at the RSV season's onset, along with individuals with specific underlying conditions. Consequently, most infants remain unprotected against RSV [26]. Therefore, protecting all infants up to 6 months of age is only possible through maternal immunization or using extended duration mAbs.

Researchers have aimed to develop extended duration mAbs that offer a more enduring protective effect against RSV than palivizumab. In 2023, the FDA approved nirsevimab for the prevention of RSV in infants and children. Nirsevimab is a recombinant human mAb with an extended half-life of 63–73 days in infants. It exhibits high potency, neutralizing RSV-A and RSV-B strains with an affinity over 50 times that of palivizumab. However, the availability of nirsevimab is currently limited [27].

A study investigated the effectiveness and safety of four monoclonal antibodies—nirsevimab, motavizumab, palivizumab, and suptavumab. The results demonstrated a significant decrease in RSV-related hospitalization, infection, and supplementary oxygen consumption. The study's findings indicated that motavizumab considerably outperformed palivizumab in reducing RSV infection in newborns, hospitalization in intensive care units (ICU), and the use of mechanical ventilation [28]. Motavizumab was an initial contender to replace palivizumab for RSV prevention, as a study with a small sample size had shown promising results [29]. However, it lacks FDA approval, possibly because of concerns related to adverse side effects [30].

#### 3.1.2. Transplacental transfer of maternal antibodies

Transplacental transfer of maternal antibodies is another approach that protects infants from RSV infection. Studies have shown that higher maternal antibody concentrations are associated with less severe RSV disease in infants [31, 32]. It also deals with the timing of safeguarding infants during their most vulnerable stage to RSV, a time when directly immunizing them (via mAbs) is difficult and costly [33]. Vaccines administered to pregnant women enhance the activity of pre-existing memory B cells. This process results in an augmented transfer of maternal antibodies, offering protection to infants during the initial five to six months of life [19]. Following this notion, the FDA recently authorized Pfizer's bivalent RSVpreF vaccine (Abrysvo) for pregnant women [34]. The vaccine is advised to be administered between the 32nd and 36th weeks of pregnancy, and typically from September to January [35]. In a phase 2b proof-of-concept trial, the RSVpreF vaccine was administered to women during the late second or third trimester. This showed a safe profile and prompted the transfer of effective neutralizing antibodies to the infants. The trial provided evidence supporting the effectiveness of the RSVpreF vaccine in pregnant women to prevent RSV-related LRTI in infants [36].

It is important to note that higher antibody levels in the mother does not necessarily guarantee increased transplacental transfer of antibodies to the foetus [37]. If the mother has high serum antibody levels due to prior natural infection, the placenta may become saturated, and vaccinating her may not provide additional benefits [32]. To address this, it could be helpful to test maternal antibody titres before vaccination to determine if vaccination is needed — though, currently, there are no official recommendations for such testing. Additionally, it remains unclear whether a woman who received an RSV vaccine during one pregnancy needs to be vaccinated again for subsequent pregnancies, as the Centers for Disease Control and Prevention (CDC) has not provided clear guidance on this [38]. Despite these uncertainties, the trial demonstrated that the RSVpreF vaccine is effective in preventing RSV-related LRTI in infants, providing significant protection during an infant's early months [36]. Table 2 offers a summarized review of important studies exploring RSV vaccination strategies for pregnant mothers.

**Table 2.** Studies on RSV Vaccination to Mothers During Pregnancy

**Study ID**	**Country**	**Study Design**	**Conclusion**
Madhi et al, 2020^39^	multicenter	Randomized control trial	The maternal RSV-F nanoparticle vaccine showed 40% efficacy against medically significant RSV lower respiratory infection in infants under 90 days, with variation between lower-middle-income countries (LMIC) and high-income countries (HIC) and had a good safety profile.
Bebia et al, 2023^40^	multicenter	Phase 2 Randomized Trial	RSVPreF3 vaccine administered to pregnant women in the late second or third trimester showed a safe profile, robust immune response, successful antibody transfer to infants, and antibody persistence for at least six months post-birth.
Kampmann et al, 2023^41^	multicenter	Randomized control trial	The trial investigated the effects of RSVpreF in over 3000 pregnant women and their infants. The vaccine demonstrated over 80% efficacy against severe illness within 90 days of birth and approximately 69% efficacy against severe illness within 180 days of birth.

A clinical trial assessed the effectiveness of the RSV F-nanoparticle (RSV-F) vaccine administered during pregnancy to prevent severe RSV infections in infants [39]. While the vaccine did not meet its pre-specified efficacy target of 30%, it demonstrated 40% efficacy in reducing RSV-related illness in the first 90 days of life, a period when many RSV cases occur [39].

The vaccine's effectiveness varied between low, lower-middle, and high-income countries. This is likely due to differences in healthcare practices (such as hospitalization rates for less severe RSV cases), breastfeeding rates, and RSV attack rates, which can influence vaccine outcomes. The vaccine was generally well-tolerated, with only mild side effects, such as localized pain at the injection site. However, further studies are needed to explore its long-term safety and potential adverse effects, particularly in pregnant women.

In another clinical trial, the RSVPreF3 vaccine was given to pregnant women in their late second or third trimesters [41]. This vaccine successfully increased neutralizing antibody levels, which were transferred from the mother to the infant. These antibodies persisted in the infants for at least six months after birth, indicating that the vaccine could provide long-term protection against RSV [40].

Additionally, a large trial involving over 3,000 pregnant women demonstrated that the RSVpreF vaccine was more than 80% effective in preventing severe RSV illness in infants during the first 90 days of life [41]. Its efficacy remained around 69% over 180 days, leading to the vaccine's fast-track approval by the FDA [41] and recognition of its potential for widespread use.

Despite these promising results, concerns remain about the safety and effectiveness of maternal RSV vaccination, especially for pre-term infants. Infants born before 28 weeks of gestation may not receive sufficient benefits from maternal vaccination, as the transfer of antibodies primarily occurs in the third trimester [37]. Consequently, preterm infants may not receive adequate protection from the vaccine.

There are also concerns about the safety of vaccination during pregnancy, including potential risks to both the mother and the infant. Addressing these concerns with clear and transparent communication is essential. Ongoing research is needed to ensure the safe and effective use of RSV vaccines in pregnant women. Despite these challenges, the RSVpreF vaccine shows significant promise in reducing RSV-related morbidity and mortality in infants, particularly those under six months of age [41]. However, continued research and open dialogue with the public are essential to maximize the benefits of this vaccine for maternal vaccination programs.

### 3.2. Maternal vs infant RSV immunization

**Figure 1.**
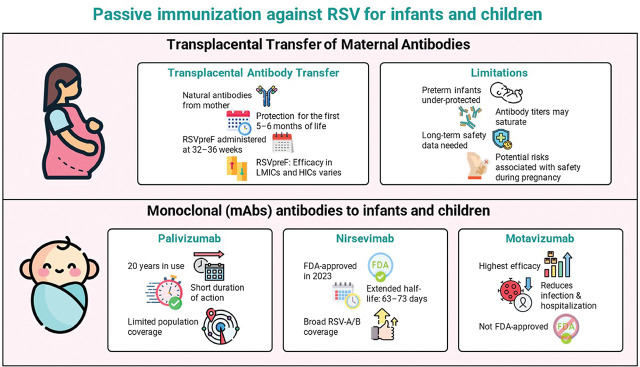
Key distinctions between maternal RSV and infant mAbs.

RSV vaccination for mothers, and mAbs for children and infants, both offer protection to children and infants, though they differ in terms of their duration of protection and the recommended number of doses, as well as their indications and contraindications. Figure 1 highlights key distinctions between maternal RSV and infant mAbs.

## 4. Factors influencing the choice of immunization strategies

The CDC emphasized that in most cases, both interventions (RSV vaccines to pregnant mothers and mAbs to children) are not necessary, and a choice between maternal vaccination or infant immunization with RSV mAb can be made based on various factors [42]. Factors including expense and insurance coverage, past experiences with RSV, access to healthcare, medical advice from healthcare professionals; and knowledge of RSV risk factors can significantly impact the decision to receive RSV immunization, whether during pregnancy as an RSV vaccine for the mother, or later for the child as mAbs.

One of the main concerns for parents receiving vaccines during pregnancy is the safety of their unborn child. Healthcare providers can play a crucial role in addressing these concerns by providing evidence-based information regarding the safety profiles of vaccines approved for use during pregnancy. Parents are particularly attentive to recommendations from healthcare providers when making decisions regarding vaccination and antibody therapy for their children. Access and affordability also influence parents' decisions regarding vaccination and antibody therapy. Parents may also face barriers to vaccination, such as limited access to healthcare facilities offering maternal vaccination, or difficulty affording the cost of vaccines or antibody therapy for their child. Healthcare providers strive to overcome these obstacles by informing families about available resources, including insurance coverage and assistance programs. RSV immunization is provided through the CDC's Vaccines for Children (VFC) program, covering eligible children at no cost [43]. For underinsured children whose health insurance may not fully cover vaccines or certain recommended vaccines, the VFC Program ensures access to vaccines at Federally Qualified Health Centers (FQHCs) or Rural Health Clinics (RHCs). These facilities in medically underserved areas have arrangements to provide immunization to eligible children.

The VFC Program enhances access to vaccinations and eliminates cost barriers by offering free vaccines to eligible children through a nationwide network of more than 37,000 public and private healthcare providers. The VFC Program covers all Advisory Committee on Immunization Practices (ACIP)-recommended and CDC-approved vaccines for children aged 18 and younger, protecting against 19 diseases, including RSV, Mpox, MMR, and Meningococcal B. This has prevented an estimated 472 million illnesses and 29.8 million hospitalizations [44]. The program has saved nearly $2.2 trillion through illness prevention [45].

A study assessed RSV awareness among pregnant women and healthcare professionals, as well as attitudes toward clinical trials and routine administration of the antenatal RSV vaccine across four hospitals in South England [46]. The findings revealed very low awareness among pregnant women and midwives compared to obstetricians. Certain demographic factors influenced attitudes toward vaccination. Younger pregnant women, those at 21–30 weeks of gestation, and those with prior RSV experience were more likely to participate in the trials [46]. White British women and those at 21–30 weeks of gestation were more open to routine vaccination as well. While willingness to participate in RSV vaccination trials varied among different demographics, the overall acceptance was higher for methods that were routinely recommended. Obstetricians showed more support for both clinical trials and routine vaccinations than midwives. The study emphasized the need for education to facilitate antenatal vaccination implementation and effective public health interventions [46].

One cross-sectional study (with the objective of validating a survey tool) focused on understanding the factors influencing RSV vaccine acceptance among women of childbearing age [47]. The study introduced the “ABCDEF” scale, consisting of Advice, Burden, Conspiracy, Dangers, Efficiency, and Fear, to unravel the complexities of RSV vaccination decision-making. Notably, despite a 70% acceptance rate for a safe, effective, and free RSV vaccine, a noteworthy proportion of the population was hesitant. The study identified “Advice” and “Fear” as pivotal determinants, underscoring the importance of effective communication from credible sources to address concerns and highlight the benefits of vaccination [47].

Another study conducted focused on analysing attitudes toward maternal RSV vaccines among pregnant and lactating individuals in Kenya [48]. The study found an association between first pregnancy and higher vaccine hesitancy among both pregnant and lactating individuals. This raises the possibility that people who are pregnant for the first time may have doubts about maternal vaccinations, and that this may affect their readiness to accept them. This study also found that social norms significantly influenced vaccine hesitancy among lactating individuals. This highlights the importance of cultural and societal influences on vaccine acceptance, emphasizing the need for tailored strategies to address community-specific concerns and perceptions [48].

## 5. Conclusion

Despite years of investigation, a dedicated RSV vaccine for children remains unavailable. However, recent FDA approval of maternal RSV vaccines has shifted the paradigm, as these vaccines provide longer-duration protection to infants compared to mAbs. Current guidelines recommend that if a mother receives the RSV vaccine during pregnancy, the infant from that pregnancy may not require mAbs. However, beyond infancy, these guidelines do not provide clarity on dosage adjustment if the mAb is administered. Additionally, there is no indication of whether antibody titers should be measured in infants whose mothers received the RSV vaccine during pregnancy before deciding to administer mAb.

The decision-making process for parents choosing between the maternal RSV vaccine or mAbs for their children is influenced by various factors, including cost-effectiveness, safety concerns, and healthcare recommendations. Further clinical research is necessary to comprehensively compare the safety, efficacy, cost-effectiveness, and disease burden between infants whose mothers received the RSV vaccine and those whose mothers received mAbs. This study will contribute to a more informed and evidence-based approach to RSV immunization strategies for infants and children.
